# Nobiletin Intake Attenuates Hepatic Lipid Profiling and Oxidative Stress in HFD-Induced Nonalcoholic-Fatty-Liver-Disease Mice

**DOI:** 10.3390/molecules28062570

**Published:** 2023-03-12

**Authors:** Zunli Ke, Chaowen Fan, Jun Li, La Wang, Haiyang Li, Weiyi Tian, Qi Yu

**Affiliations:** Basic Medical School, Guizhou University of Traditional Chinese Medicine, Guiyang 550025, China; kezunli031@gzy.edu.cn (Z.K.);

**Keywords:** oxidative stress, lipid accumulation, NAFLD, lipidomic, Nrf2

## Abstract

Nobiletin (NOB) is a naturally occurring compound, commonly found in citrus peel, that shows hepatoprotective and lipid-reducing effects. However, the lipid biomarkers and the potential improvement mechanisms have not been adequately explored. Therefore, we investigated the ameliorative effect and the molecular mechanism of NOB on NAFLD induced by a high-fat diet in mice. The results showed that supplementation with NOB over 12 weeks markedly improved glucose tolerance, serum lipid profiles, inflammatory factors, hepatic steatosis, and oxidative stress. These beneficial effects were mainly related to reduced levels of potential lipid biomarkers including free fatty acids, diacylglycerols, triacylglycerols, and cholesteryl esters according to hepatic lipidomic analysis. Twenty lipids, including DGs and phosphatidylcholines, were identified as potential lipid biomarkers. Furthermore, RT-qPCR and Western blot analysis indicated that NOB inhibited the expression of lipogenesis-related factors such as SREBP-1c, SCD-1, and FAS, and upregulated the expression of lipid oxidation (PPARα) and cholesterol conversion (LXRα, CYP7A1, and CYP27A1) genes as well as antioxidation-related factors (Nucl-Nrf2, NQO1, HO-1, and GCLC), indicating that NOB intake may reduce lipid biosynthesis and increase lipid consumption to improve hepatic steatosis and oxidative stress. This study is beneficial for understanding the ameliorative effects of NOB on NAFLD.

## 1. Introduction

Polymethoxylated flavones (PMFs) are a subgroup of plant flavonoids that occur mainly in citrus fruits (*Citrus* L. Rutaceae), especially in the citrus peels. PMFs are of interest for their various biological activities, such as their anti-oxidative, anti-inflammatory, and anti-cancer properties [1]. Nobiletin (5,6,7,8,3′,4′-hexamethoxyflavone, NOB) is one of the main PMFs in citrus peel, and it exhibits a potent regulation of lipid accumulation, antidiabetic activity, hepatoprotective activity, anti-inflammatory activity, antioxidant activity, and so on [2,3]. The literature reports that NOB showed benefits for dyslipidemia in both C57BL/6 and LDL receptor-deficient (*Ldlr^-/-^*) mice fed a high-fat diet (HFD) [4,5]. NOB increased the gene expression of PPARα co-activator 1α (*Pgc1α*) in the liver, leading to an increase in fatty acid (FA) oxidation [4]. Furthermore, NOB downregulated the expression of hepatic sterol regulatory element-binding protein-1c (*Srebp-1c*) in a high-glucose-induced HepG2 cells model [6]. These regulating actions might account for the beneficial activity of NOB in attenuating hepatic triglyceride (TG) deposition and improving metabolic disorder. Although the beneficial regulation of NOB on hyperlipemia and lipid metabolism has been reported in studies using lipidomics to explore the efficacy of NOB, the lipid biomarkers responsible for the bioactivities of NOB and the amelioration of hepatic oxidative stress in a non-alcoholic fatty liver disease (NAFLD) mouse model are rare.

NAFLD is a manifestation of hepatic metabolic syndrome. It is always characterized as “simple” steatosis (fatty liver) with a potentially progressive inflammatory phenotype of nonalcoholic steatohepatitis (NASH) that can progress to cirrhosis and/or hepatocellular cancer [7]. It affects approximately a quarter of the population worldwide, and its prevalence continues to increase in the context of the growing obesity epidemic, especially in western countries [8]. In China, the prevalence of NAFLD has increased significantly from 18 to 29% within a decade. It is predicted that China will soon have the highest growth in the prevalence of NAFLD [9]. The prevalence of NAFLD can reach 90–95% in obese individuals and affects up to 70% of patients with type 2 diabetes [10]. In addition, patients with NAFLD are at higher risk of cardiovascular diseases. Because there are no licensed therapies, NAFLD is predicted to become the most common indication leading to liver transplantation [11]. Therefore, approved therapeutic strategies for this disease are urgently warranted.

NAFLD is characterized by the excessive deposition of lipids in hepatocytes, and this occurs concurrently with increased lipotoxicity from high levels of free fatty acids (FFA), free cholesterol (TC), and other lipid metabolites, which could lead to mitochondrial dysfunction with oxidative stress and generation of excessive reactive oxygen species (ROS) [12]. Oxidative stress is regarded as a main inducer in the pathophysiology of inflammatory chronic liver diseases, including NAFLD. Chronic lipid disturbance is strongly associated with impairment of the balance between oxidants and antioxidants, which affect metabolism-related organelles, leading to cellular lipotoxicity, mitochondrial dysfunction, and lipid peroxidation [13,14]. Increased oxidative stress also activates hepatocyte stress pathways, leading to inflammation and causing the progression of NASH. The nuclear factor-E 2-related factor-2 (*Nrf2*) pathway is a pivotal component to protect against oxidative-stress-induced injury by upregulating its target factors such as heme oxygenase 1 (*Ho-1*), glutamate cysteine ligase catalytic subunit (*Gclc*), NAD(P)H:quinone oxidoreductase (*Nqo1*), and glutathione S-transferase A2 (*Gsta2*), contributing to the restoration of normal lipid metabolism. Thus, alleviating hepatic oxidative stress by the Nrf2 pathway emerges as an effective therapy to improve NAFLD development and progression [13].

Lipidomics is an effective and sensitive strategy that is capable of exploring biological responses by investigating lipid composition and identifying lipid biomarkers at a molecular level [15,16]. Currently, lipidomic analysis has been applied to evaluate the beneficial effects of natural compounds [17,18,19]. For example, lipidomic analysis revealed that tangeretin intake showed cholesterol-lowering effects and this function was related to lowering the levels of FFA, diacylglycerols (DGs), TGs, ceramides (Cers), and cholesteryl esters (CEs) [18]. Moreover, lipidomic analysis showed that the lipid-lowering effects of arabinoxylan, that had been attributed to the decreasing levels of some FFA, 12α-hydroxylated bile acids, and carnitines (CARs) on type 2 diabetic rats, were in fact due to an increase in lysophosphatidylcholine (LPC) levels. In addition, the underlying molecular mechanism of the beneficial effects of dietary ω-3 polyunsaturated fatty acids was also investigated by lipidomic analysis [20]. Based on the aforementioned studies, lipidomics has become an important tool of mechanistic exploration in nutrition and medicinal research. 

In this work, we hypothesize that NOB has beneficial effects on hepatic lipid metabolism and oxidative stress as well as the associated metabolic dysfunction in NAFLD mice. Therefore, we combined biochemical and lipidomic analysis to investigate the effect and the underlying mechanisms of the regulation of lipid metabolism and oxidative stress after supplementing with NOB in a HFD-induced NAFLD mouse model.

## 2. Results

### 2.1. Effects of NOB on Body Weight Gain, Food Intake, and GTT in HFD-Induced NAFLD Mice

**Figure 1:**
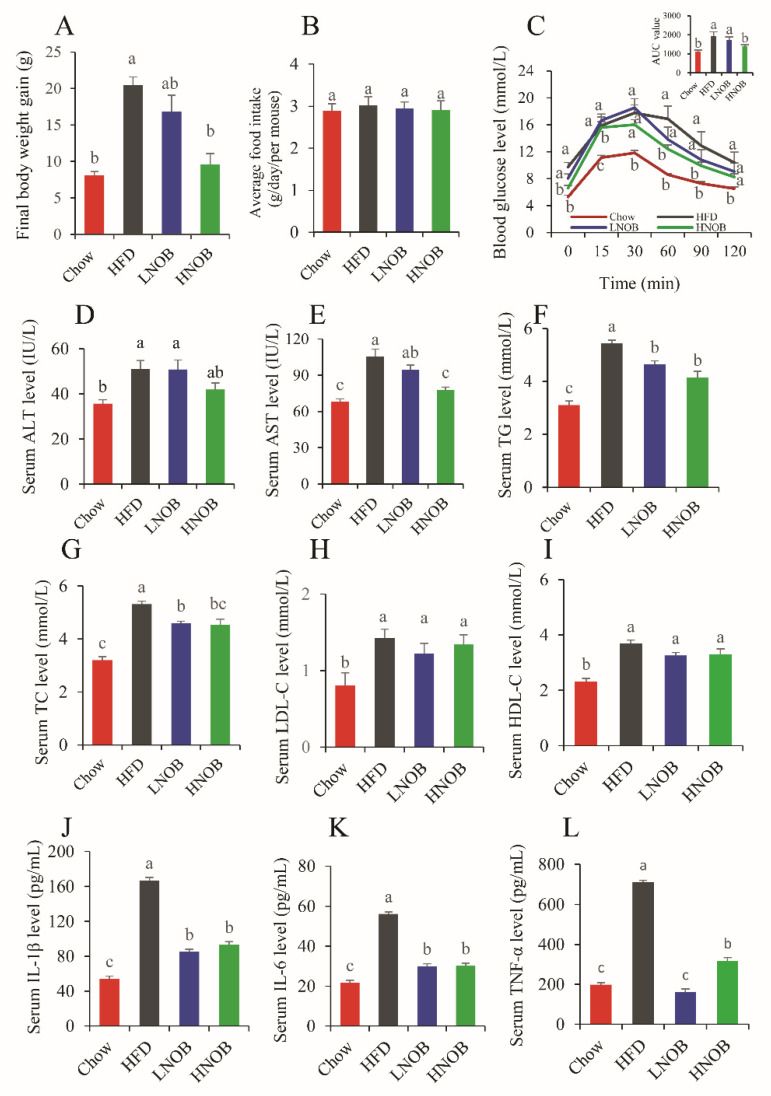
NOB inhibited body weight gain and ameliorated glucose tolerance, serum lipid profiles, as well as inflammatory factors in HFD-induced mice. (**A**) Final body weight gain. (**B**) Food intake. (**C**) GTT and AUC. (**D**) Serum ALT level. (**E**) Serum AST level. (**F**) Serum TG level. (**G**) Serum TC level. (**H**) Serum LDL-C level. (**I**) Serum HDL-C level. (**J**) Serum IL-1β level. (**K**) Serum IL-6 level. (**L**) Serum TNF-α. Data are shown as mean ± SD (*n* = 8). Data with different letters (a, b, c) represent significant difference (*p* < 0.05), whereas bars with the same letter correspond to values that exhibit no significant differences. In the case where two letters are present on top of the bars, each letter should be compared separately with the letters of other bars to determine whether the values show significant differences.

HFD-fed mice exhibited a significantly higher body weight gain when compared to the Chow group mice, but a dietary supplementation of 0.1%NOB (LNOB) and 0.2%NOB (HNOB) could reduce the body weight gain. HNOB significantly inhibited the body weight gain induced by a high-fat diet (Figure 1A). It was also found that daily NOB supplementation showed no significant change in food intake when compared with the HF-diet group (Figure 1B).

A long-term HFD is associated with obesity, dyslipidemia, and glucose intolerance. As shown in Figure 1C, the FBG in the HFD group mice was higher than that in the Chow group, while a 12-week dietary supplementation with NOB decreased the FBG and the HNOB group mice showed a significant reduction. During the GTT, HNOB group mice had lower glucose levels at 15 min, 60 min, and 90 min. In addition, the HNOB-supplementation group also showed significantly lower AUC values when compared with the HFD group. However, the LNOB-supplementation mice only showed a lower glucose level at 15 min (Figure 1C). In summary, dietary supplementation with NOB alleviated abnormal glucose levels caused by HFD feeding.

### 2.2. Effect of Dietary NOB on Serum Biochemistry in HFD-Induced NAFLD Mice

As shown in Figure 1D, E, the HFD group mice had obviously higher ALT and AST levels, indicating the induction of liver injury by the HFD. HNOB supplementation decreased the AST level but not the ALT level, after 12 weeks of feeding. Moreover, Figure 1F–I show that the HFD group mice had dyslipidemia, which was characterized by the increasing serum TC, TG, and LDL-C levels when compared with the Chow group mice. Interestingly, dietary supplementation with NOB significantly reduced serum TG and TC levels but did not affect the serum LDL-C and HDL-C levels.

NAFLD is always accompanied by inflammation. Therefore, we analyzed the inflammatory factors in serum. As shown in Figure 1J–L, the serum IL-1β, IL-6, and TNF-α concentrations were significantly increased in HFD group mice (compared with the Chow group). However, NOB supplementation for 12 weeks obviously reduced the serum levels of IL-1β, IL-6, and TNF-α.

### 2.3. Effect of NOB Supplementation on Hepatic Lipid Accumulation in HFD-Induced NAFLD Mice

**Figure 2:**
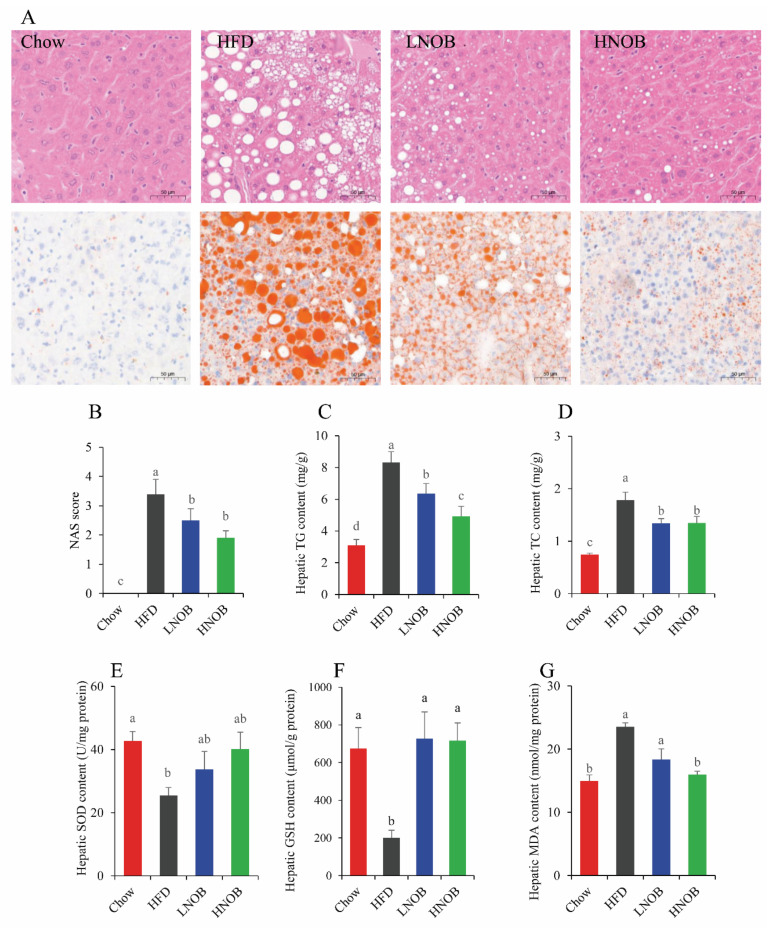
NOB ameliorated hepatic steatosis and oxidative stress in HFD-induced mice. (**A**) HE and Oil Red O staining (×200). (**B**) NAS score. (**C**) TC content in the liver. (**D**) TG content in the liver. (**E**) Hepatic SOD content. (**F**) Hepatic GSH content. (**G**) Hepatic GSH content. Data are shown as mean ± SD (*n* = 8). Data with different letters (a, b, c) represent significant difference (*p* < 0.05), whereas bars with the same letter correspond to values that exhibit no significant differences. In the case where two letters are present on top of the bars in E, each letter should be compared separately with the letters of other bars to determine whether the values show significant differences.

As shown in Figure 2A, the HFD group mice showed moderate to obvious macrovesicular steatosis shown by hepatic H&E staining, when compared with the Chow group, whereas NOB administration improved hepatocyte steatosis. In addition, the ORO staining also showed an excessive lipid droplet accumulation in the livers of HFD group mice in contrast to the Chow group, and NOB intake decreased the size of these lipid droplets (Figure 2A). Figure 2B shows that the HFD caused higher NAS (compared with that of the Chow group), while both LNOB and HNOB group mice demonstrated lower NAS (compared with that of the HFD group). Overall, the histological investigation found that excessive lipid deposition and the degree of liver steatosis were both improved by NOB supplementation. Mice in the HFD group also showed significantly higher TG and TC contents in the liver (compared with the Chow group), indicating obvious hepatic lipid deposition. Expectedly, TG and TC levels were significantly reduced (compared with the HFD group) in the LNOB and HNOB groups (Figure 2C,D). Taken together, these results provided evidence that NOB treatment might reduce lipid accumulation in the liver induced by a HFD, thereby exerting an ameliorative effect on hepatic steatosis.

### 2.4. Effect of NOB Supplementation on Hepatic Oxidative Damage in HFD-Induced NAFLD Mice 

Excessive hepatic lipid accumulation leads to hepatic oxidative damage. As shown in Figure 2E–G, HFD mice exhibited a lower content of the antioxidant substances SOD and glutathione (GSH), and a higher content of peroxidation products MDA when compared with the Chow group. In contrast, NOB administration improved the hepatic antioxidant capacity of mice including elevating the GSH concentration and reducing the MDA concentration. These results indicated that NOB treatment might ameliorate the oxidative injury of the liver caused by HFD feeding. 

### 2.5. Lipidomic Analysis after NOB Supplementation

**Figure 3:**
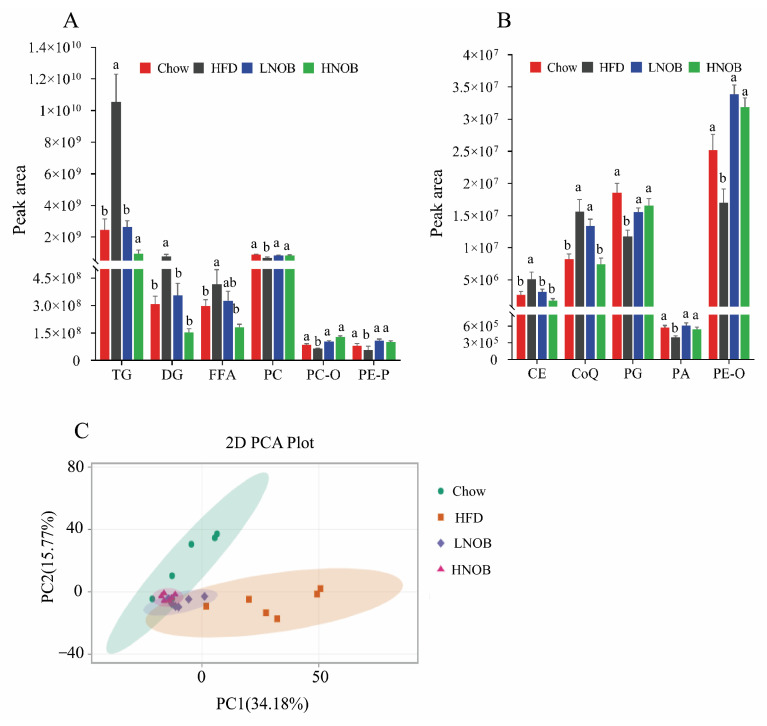
Lipidomic analysis of liver samples. The relative intensity of different lipid compositions in liver tissues (**A**,**B**). The PCA score plots of lipid profiling in liver tissues among four groups (**C**). Data are shown as mean ± SD (*n* = 6). Data with different letters (a, b) represent significant difference (*p* < 0.05), whereas bars with the same letter correspond to values that exhibit no significant differences. In the case where two letters are present on top of the bars in A, each letter should be compared separately with the letters of other bars to determine whether the values show significant differences.

In order to explore the effect of NOB on hepatic lipid biomarkers, the species-level analyses of lipids from Chow, HFD, LNOB, and HNOB groups were analyzed according to the lipidomic approach. A total of 1276 lipid metabolites were identified from the four group of mice, including 17 eicosanoids, 39 FFAs, 280 TGs, 101 DGs, 12 phosphatidic acid (PAs), 107 phosphatidylcholines (PCs), 29 etherphosphatidylcholines (PC-Os), 84 phosphatidylethanolamines (PEs), 78 etherphosphatidylethanolamines (PE-Os), 57 phosphatidylethanolamine-based plasmalogens (PE-Ps), 32 phosphatidylglycerols (PGs), 43 phosphatidylinositols (PIs), 51 carnitines (CARs), 14 cholesteryl esters (CEs), 40 ceramides (Cers), 3 coenzyme Qs (CoQs), and others. As shown in Figure 3A,B, the total contents of TG, DG, FFA, CoQ, and CE in the HFD group were obviously higher, whereas the total contents of PC, PC-O, PE-P, PG, PA, and PE-O were significantly lower when compared with Chow group mice. However, the contents of these lipids were significantly reversed after NOB administration (compared with HFD group). On the basis of these results, consumption of 0.1% and 0.2% NOB could effectively ameliorate the disorder of lipid metabolism caused by a HFD in mice. In addition, principal components analysis (PCA) was used to analyze the lipid composition among the four groups. From Figure 3C, it is evident that the HFD group is distinctly separated from the Chow group. As expected, the NOB supplementation groups, especially the HNOB group, are separated away from the HFD group and close to the Chow group, implying that the NOB supplementation might ameliorate the disturbances of lipid metabolisms toward the normal condition.

### 2.6. NOB Supplementation Changed the Important Differential Lipid Species in The Liver

**Figure 4:**
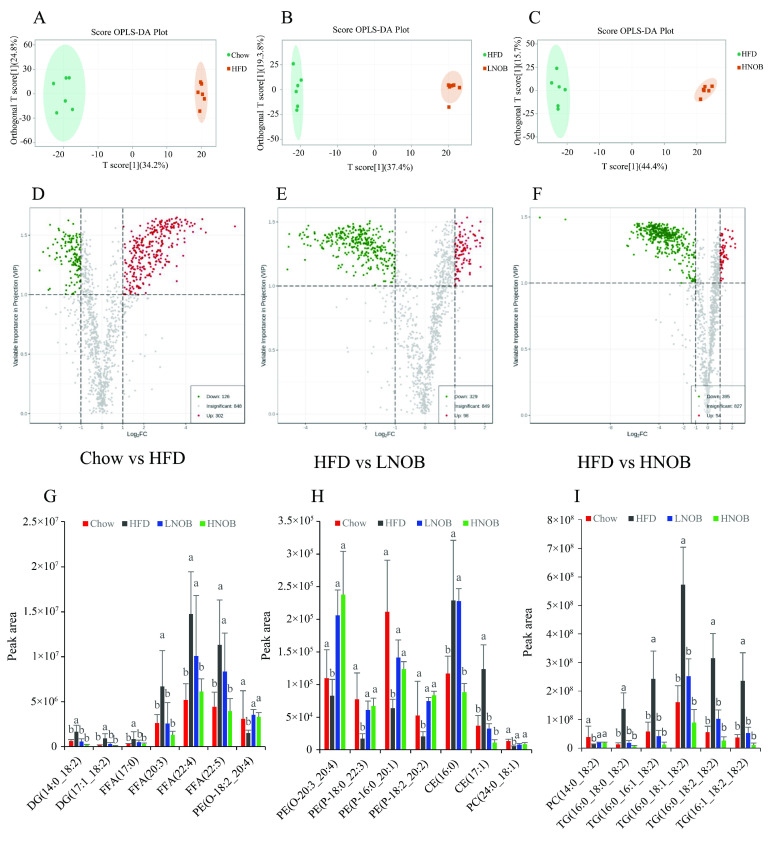
Analysis of markedly differential hepatic lipid species in Chow vs. HFD, HFD vs. LNOB, and HFD vs. HNOB. The OPLS-DA scores (**A**–**C**) and volcano plot (**D**–**F**) analysis of Chow vs. HFD, HFD vs. LNOB, and HFD vs. HNOB, respectively. The obviously differential lipid species were selected out by using the criteria of FC ≥ 2 or ≤ 0.5 and VIP ≥ 1 in the volcano plot, where the obviously differential lipid species were shown using a red (up) or green (down) dot, whereas a gray dot represented no significant difference of lipid species. (**G**–**I**) The potential lipid biomarkers in response to the beneficial effects of NOB in HFD-induced mice were selected out by using the criteria of fold change ≥ 2 or ≤ 0.5 and VIP ≥ 1. Data are shown as mean ± SD (*n* = 6). Data with different letters (a, b) represent significant difference (*p* < 0.05), whereas bars with the same letter correspond to values that exhibit no significant differences.

To obtain a better understanding of the separation between different groups, orthogonal partial least squares discriminant analysis (OPLS-DA) was conducted to estimate the alterations in global lipids. Three separate multivariate OPLS-DA models (HFD group vs. Chow group, LNOB group vs. HFD group, and HNOB group vs. HFD group) were generated. From Figure 4A–C, we observed an obvious separation of the OPLS-DA score plot shown in the Chow vs. HFD, HFD vs. LNOB, and HFD vs. HNOB, respectively, indicating that lipid metabolic disorders were induced by HFD feeding and improved by NOB supplementation. Volcano plot analysis was employed to identify lipid biomarker candidates accounting for this distinction using the following criteria: FC (Fold change) ≥ 2 or ≤ 0.5 and variable importance in the projection (VIP) ≥ 1, *p <* 0.05. As illustrated in Figure 4D, 428 differentially regulated lipid species were screened out in the Chow group vs. the HFD group model. Among the 428 identified lipids, the levels of 302 lipid species were observably elevated and 126 lipid species were significantly reduced. Notably, 427 lipids were obviously different between HFD and LNOB groups and 449 lipids were obviously different between the HFD and HNOB groups as shown in Figure 4E,F, respectively, and most of them were remarkably downregulated. Taken together, these findings revealed that NOB supplementation can effectively improve the disturbance in lipid metabolism induced by a HFD. Furthermore, the differences in the levels of the possible lipid biomarkers among four groups are shown in Figure 4G–I using the following criteria (VIP > 1, fold change ≥ 2 or ≤ 0.5, *p* < 0.5). These significantly changed lipid species including 2 DGs, 4 FFAs, 2 PE-Os, 3 PE-Ps, 2 CEs, 2PCs, and 5 TGs were screened out from the Chow, HFD, LNOB, and HNOB groups. As illustrated in Figure 4G–I, the levels of DG (14:0_18:2), DG (17:1_18:2), FFA (17:0), FFA (20:3), FFA (22:4), FFA (22:5), CE (16:0), CE (17:1), PE (O-18:2_20:4), PE(O-20:3_20:4), PE (P-18:0_22:3), PE (P-16:0_20:1), PE (P-18:2_20:2), PC (24:0_18:1), PC (14:0_18:2), TG (16:0_18:0_18:2), TG (16:0_16:1_18:2), TG (16:0_18:1_18:2), TG (16:0_18:2_18:2), and TG (16:1_18:2_18:2) were markedly changed in the HFD group (compared with the Chow group), implying that HFD feeding led to a significant change in the lipid profile. In contrast, the levels of these lipids were improved after 12 weeks of NOB supplementation. Therefore, the results imply that these lipid markers might be considered as potential biomarkers responsible for the amelioration effects of NOB on fatty liver induced by a HFD.

### 2.7. Pathway Analysis

**Figure 5:**
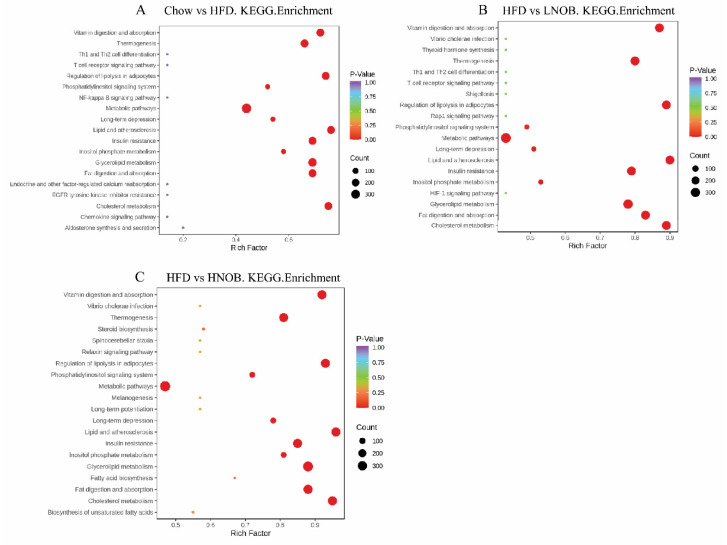
Lipid metabolic pathway analysis based on markedly differential lipid species in Chow vs. HFD (**A**), HFD vs. LNOB (**B**), and HFD vs. HNOB (**C**). Degree of enrichment was analyzed by a rich factor, *p*-value, and the number of lipid metabolites enriched in each pathway. The size of the bubble shows the number of markedly differential lipid species that are enriched in this pathway, and the point with a different gradation of color represents the scope of the *p*-value. A higher rich factor value stands for a higher degree of enrichment, and a lower *p*-value represents a more significant degree of enrichment.

Pathway enrichment analysis was carried out to investigate the most relevant metabolic pathways associated with lipid metabolism. As exhibited in Figure 5A, the mice fed with the HFD had a markedly affected vitamin digestion and absorption, thermogenesis, regulation of lipolysis in adipocytes, lipid and atherosclerosis, insulin resistance, glycerolipid metabolism (GL), fat digestion and absorption, and cholesterol metabolism when compared with the Chow group. However, LNOB and HNOB supplementation improved these changes (Figure 5B,C). These data suggest that NOB greatly improved lipid metabolism in NAFLD mice via these metabolic signaling pathways.

### 2.8. Effect of NOB Supplementation on the Expression of Genes and Proteins Involved in Hepatic Lipid Metabolism and Oxidative Stress

**Figure 6:**
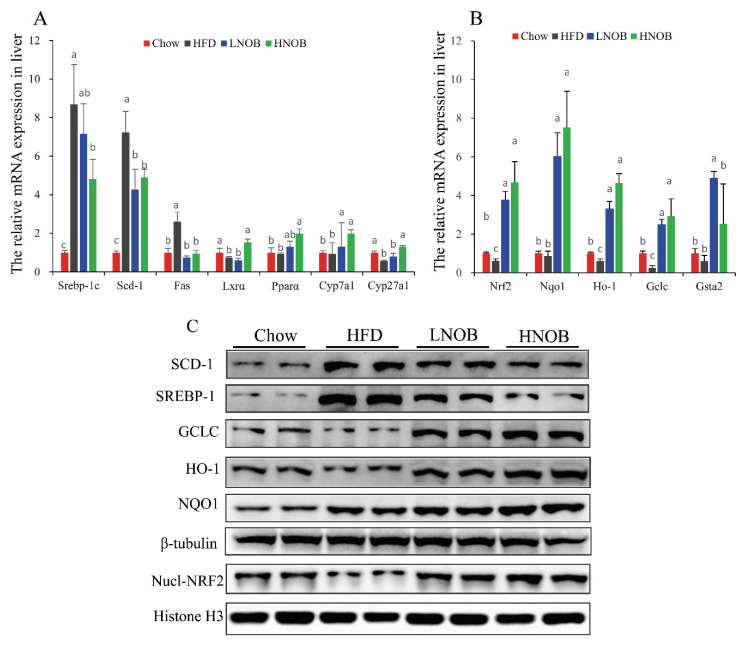
The effects of NOB supplementation on the expression of lipid metabolism and Nrf2-pathway-related factors in HFD-induced mice. The mRNA expression of hepatic lipid metabolism genes (**A**) and anti-oxidation genes (**B**). Data are shown as the mean ± SD (*n* = 4). Data with different letters (a, b, c) represent significant differences (*p* < 0.05), whereas bars with the same letter correspond to values that exhibit no significant differences. In the case where two letters are present on top of the bars in A, each letter should be compared separately with the letters of other bars to determine whether the values show significant differences. (**C**) Representative immunoblots for SCD-1, SREBP-1, GCLC, HO-1, NQO1, β-tubulin, Nucl-NRF2, and Histone-H3.

In order to investigate the potential mechanism related to the amelioration by NOB of lipid metabolism and oxidative stress in the liver tissues, we further analyzed the expression of genes and proteins associated with lipid metabolism and oxidative stress. Figure 6A demonstrates that the lipid-metabolism-related genes including *SREBP-1c*, *fatty acid synthases (Fas), Scd1*, *liver X receptor (Lxrα)*, *peroxisome proliferator-activated receptor α (Pparα)*, *cholesterol 7alpha-hydroxylase gene (Cyp7a1)*, and *sterol 27-hydroxylase (Cyp27a1)* were significantly disrupted in the HFD group mice (compared with Chow group), whereas the mRNA expression of *Srebp-1c*, *Fas,* and *Scd1* in both NOB supplementation groups, and especially in the HNOB treatment group, was significantly lower. In addition, the levels of lipolysis gene *Pparα* were obviously increased (compared with HFD group) in the NOB group. Moreover, HNOB supplementation remarkably elevated (in comparison with the HFD group) the expression level of *Lxrα*, *Cyp7a1,* and *Cyp27a1*, which were involved in cholesterol metabolism. As shown in Figure 6B, supplementation of NOB significantly upregulated the expression of *Nrf2* and its target genes, such as *Ho-1*, *Nqo1*, *Gclc*, and *Gsta2*, which are involved in protecting cells against oxidative stress. Furthermore, the protein levels of SCD-1, SREBP-1, Nrf2, GCLC, HO-1, and NQO1 were assessed by WB analysis. Similar to the results of the mRNA expression analysis, the HFD also elevated SCD-1 and SREBP-1 protein levels in comparison with the Chow group. In contrast, the elevated expression of these protein expressions was reduced by LNOB and HNOB supplementation (Figure 6C). Additionally, the Nucl-NRF2 protein level and those of its target proteins, such as GCLC, HO-1, and NQO1, were also significantly increased in both of the NOB-treated groups (Figure 6C).

## 3. Discussion

Previous studies have reported that abnormal hepatic alterations in lipids are crucial pathophysiological hallmarks of NAFLD and higher levels of hepatic lipids lead to oxidative stress and overproduction of ROS [21]. Citrus PMFs are attracting increasing attention due to their beneficial efficacy in the improvement of metabolic syndrome and lipid metabolism [4,5,22,23]. Despite previous studies demonstrating the lipid-reducing effect of NOB [4,6], the mechanism of NOB regulation of lipid homeostasis remains inadequately studied. Therefore, in the present work, hepatic lipidomics, RT-qPCR, and WB were applied to provide new insights into lipid metabolism in NAFLD after NOB intervention. We found that NOB supplementation ameliorated the disturbances in blood glucose, serum lipids, inflammatory factors, hepatic lipid accumulation, steatosis, and oxidative stress in HFD-induced NAFLD mice.

A previous study reported the lipid-lowering effect of NOB, but NOB had no effect on body weight [24]. However, we found that 0.2%NOB supplementation could inhibit body weight gain, which might be due to the fact that the dose of NOB in the present work was higher. The level of serum AST was significantly decreased and showed a recovery trend back toward the Chow group after 0.2%NOB supplementation, demonstrating that NOB has a good safety profile, which had been described in previous work [25]. In addition, 0.2%NOB supplementation exhibited an ameliorative effect on hepatic oxidative injury induced by the HFD. The literature also reports that NOB administration can improve glucose concentration and serum lipid levels, as well as inflammatory factors, in rats or mice fed a western diet [24,26]. Our data also show that NOB-supplemented groups of mice exhibited significantly lower serum TC, TG, IL-1β, IL-6, and TNF-α levels, which had been elevated by HFD feeding. Our results are consistent with these previous studies.

In current work, we found that NOB intake could reduce hepatic TG and TC content and improve hepatic steatosis by lowering the expression of genes related to lipogenesis (*Srebp-1c*, *Scd-1,* and *Fas*) and increasing the expression of genes involved in lipid oxidation (*Pparα*). Moreover, the expression levels of SREBP-1c and SCD-1 proteins were also significantly reduced in NOB-treatment mice. Mulvihill et al. found that NOB prevented the hepatic lipid load in *Ldlr^-/-^* mice, mainly by enhancing the expression of peroxisome-proliferator-activated receptor gamma coactivator-1 alpha (*Pgc-1α*) and increased fatty acid β-oxidation [4]. In addition, NOB has been reported to inhibit lipogenesis via activation of the AMPK pathway in high-glucose-induced hepatic lipid accumulation in HepG2 cells [6]. These results imply that NOB could improve dyslipidemia and hepatic steatosis partly due to reduced hepatic lipid synthesis and increased lipid consumption.

As we all know, oxidative stress is regarded as a main cause of the progression of NAFLD. Excessive hepatic lipid accumulation leads to an imbalance between oxidant and antioxidant systems, leading to excessive ROS generation, mitochondrial dysfunction, lipid peroxidation, and reduced antioxidant enzymes [13]. Therefore, ameliorating hepatic oxidative stress might improve NAFLD. Nrf2 is a key factor in counteracting oxidative-stress-induced damage, contributing to the restoration of normal lipid metabolism [27]. A previous report has shown that NOB supplementation enhanced SOD activity both in plasma and hepatic tissue, along with concurrent downregulated liver NADPH oxidase subunit gp91^phox^ expression in HFD-fed rats [26]. In contrast, our research showed that NOB supplementation reduced the content of MDA and enhanced the activity of GSH by up-regulating the expression levels of antioxidant factors of NRF2 and its targeted factors HO-1, NQO1, and GSTA2 in the liver tissues, thereby ameliorating the hepatic oxidative stress induced by the HFD. To our knowledge, this is the first report that NOB might improve hepatic oxidative injury through the Nrf2 pathway in a NAFLD mouse model.

Long-term dysfunction in lipid metabolism is a hallmark of metabolic syndromes, such as NAFLD [7]. Lipid metabolism disorders, such as excess hepatic TG deposition, can cause the development of hepatic steatosis [8]. In the present study, a lipidomic study was applied to define the types and amounts of lipids that changed in an NAFLD mouse model, thereby aiming to understand the beneficial effects of NOB intervention. The results of hepatic lipidomic analysis showed that the total contents of TG, DG, FFA, and CE were significantly higher in the HFD group (compared to the Chow group), which was also found in previous reports [18]. As expected, NOB administration was associated with lower levels of these lipid species in lipidomic profiles. Higher levels of FFAs can not only cause abnormal hepatic TG storage but can also be converted into lipid intermediates, such as DGs, affecting normal cellular functions. It has been reported that DG intermediates are lipotoxic and are considered as crucial components for accelerating the deterioration of NAFLD [28]. Expectedly, NOB treatment remarkably lowered the deposition of FFAs, TGs, CEs, and DGs, indicating the improvement effect of NOB on hepatic steatosis. The metabolism of TGs, DGs, and FFAs is tightly associated with the factors involved in lipogenesis (SREBP-1c, SCD-1, and FAS) and lipid oxidation (PPARα). *Srebp-1c* is a key transcription factor in lipogenesis, regulating the expression of its targeted genes, *Scd-1* and *Fas*, which mediate the synthesis of TG and fatty acids [29]. In our work, NOB intake inhibited the expression levels of SREBP-1c, SCD-1, and FAS to reduce TG production and fatty acid biosynthesis, which might contribute to the lower content of the TGs, DGs, and FFAs in lipidomic profiles in the liver and the lower levels of hepatic TG. In addition, NOB supplementation increased the mRNA level of Pparα, which participates in enhancing fatty acid oxidation. Therefore, the reduced content of TGs, DGs, and FFAs may have also had a close relationship with the increase in lipid oxidation after NOB administration. Furthermore, activation of SCD-1 is also involved in the synthesis of CEs [30]. The lower levels of CEs are also observed in *Scd-1*-deficient mice [31], indicating that inhibition of SCD-1 expression might contribute to the lower level of CEs after NOB intake. A previous study reported that activation of hepatic Lxrα increased the conversion of cholesterol into bile acids by upregulating downstream genes such as *Cyp7a1* and *Cyp27a1* [32]. Our results demonstrate that NOB supplementation upregulated the genes expression of *Cyp7a1* and *Cyp27a1*, which promote cholesterol conversion into bile acids, while reducing the hepatic TC content. These results are consistent with the findings of the pathway analysis, that NOB intake has an obvious effect on cholesterol metabolism. These data reveal that NOB might exert a cholesterol-reducing benefit on mice fed an HF diet and this pathway may also be beneficial for the improvement of NAFLD. Collectively, the improvement effect of NOB on the NAFLD mouse model may be attributed to the regulation of lipid synthesis and oxidation, thereby affecting liver lipidomic profiles.

Previous findings have proposed that glycerophospholipids (GPs), such as PE and PC, played key roles in the progression of NAFLD [17,19]. In our study, several GPs, including PE (O-18:2_20:4), PE(O-20:3_20:4), PE (P-18:0_22:3), PE (P-16:0_20:1), PE (P-18:2_20:2), PC (24:0_18:1), and PC (14:0_18:2), were significantly increased after NOB supplementation (compared with the HFD group). PC is a hydrophilic lipid and is associated with packaging and exporting neutral lipids (e.g., TGs) as VLDL. Thus, the impairment of PC biosynthesis inhibits the synthesis and secretion of VLDL, which contributes to the development of NAFLD [33]. In addition, lipidomic studies of the human liver have demonstrated that NAFLD is associated with a decrease in PC [34]. Similar results were found in our study; the PC was significantly decreased in the HFD group, but NOB treatment increased the PC level. This may also partly contribute to the lower TG levels in liver tissues reported in the lipidomic studies.

## 4. Materials and Methods

### 4.1. Chemicals and Reagents

NOB (purity ≥ 98%) was obtained from Shanghai Yuanye Bio-Technology Co., Ltd. (Shanghai, China). Paraformaldehyde, urethane, Triton, and Tris-HCl were purchased from Solarbio Science & Technology Co., Ltd. (Beijing, China). Trizol reagent was obtained from Takara (Beijing, China). Hematoxylin and eosin (H&E), the BCA protein assay, Oil Red O (ORO), and total nuclear protein extraction and protein extraction kits were purchased from Beyotime Biotechnology (Beijing, China). The Fast Quantity RT kit was obtained from GenStar Biotechnology (Beijing, China). Commercial kits used to analyze the TC, TG, high-density lipoprotein cholesterol (HDL-C), low-density lipoprotein cholesterol (LDL-C), alanine transaminase (ALT), aspartate transaminase (AST), interleukin-1 beta (IL-1β), interleukin-6 (IL-6), and tumor necrosis factor-alpha (TNF-α) levels were purchased from Sino Best Biological Technology (Shanghai, China). HPLC-grade acetonitrile (ACN), methanol (MeOH), isopropanol (IPA), and tert-butyl methyl ether (MTBE) were purchased from Merck (Darmstadt, Germany). HPLC-grade formic acid (FA) was purchased from Sigma (Sigma Aldrich, USA). The antibodies against Nucl-NRF2 (ab62352), HO-1 (ab219360), NOQ1 (ab80588), GCLC (ab207777), stearoyl-CoA desaturase-1 (SCD-1) (ab236868), SREBP-1 (ab28481), β-tubulin (ab6046), and histone-H3 (ab1791) were purchased from Abcam.

### 4.2. Animal Experiments

The procedure for the animal experiments was authorized by the Institutional Animal Care and Ethical Committee of Guizhou University of Traditional Chinese Medicine (Approval no: 20210015, Guizhou, China). Male wild-type C57BL/6J mice aged 6–8 weeks were purchased from Changsha Tianqin Biotechnology Co., Ltd. (Changsha, Hunan, China). Mice were housed in a barrier system with a 12/12 h light–dark cycle, a regular temperature (23–26 °C), and 40–70% humidity. The mice had access to diet and water ad libitum throughout the experiment.

After 1-week adaptive feeding, mice were divided into four groups: Chow group (Research Diets, D12450B), high-fat diet group (HFD) (Research Diets, D12492), HF diet supplemented with 0.1%NOB (LNOB) (*w*/*w*) (*n* = 8) and HF diet supplemented with 0.2%NOB (HNOB) (*w*/*w*) (n = 8). The detailed components of the experimental diets are shown in Table 1. The dose of NOB used in the present study was informed by previous works [22,23].

At the end (12 weeks) of the animal experiment, all mice were anesthetized using 20%urethane after fasting for 12 h. Blood was obtained from the heart, and thereafter, the supernatant serum samples were collected by centrifuging at 1000 g for 20 min at 4 °C. The liver samples were rapidly frozen using liquid nitrogen and then stored at −80 °C for further analysis. A small part of liver tissues was fixed in 4% buffered formalin for histological analysis.

### 4.3. Glucose Tolerance Test (GTT)

The GTT was carried out as previously reported [22]. Briefly, the mice were fasted for 12 h and, thereafter, the fasting glucose blood (FBG) was sampled from the tail vein (0 min). The blood glucose blood concentration at the time points of 15, 30, 60, 90, and 120 min was tested after an intraperitoneal injection of 1 g per kg of body weight of glucose (Sinopharm Chemical Reagent Co., Ltd., Shanghai, China). 

### 4.4. Biochemical Analysis and Hepatic Histological Analysis

The serum levels of TC, TG, HDL-C, LDL-C, ALT, AST, IL-1β, IL-6, and TNF-α were analyzed using serum biochemistry kits following the manufacturer’s specification. The procedures for the H&E and ORO-staining experiments were carried out as described in our previous study [22], and the images were captured using a Zeiss Axio Imager microscope. In addition, the NAFLD activity score (NAS) was used to measure the severity of NAFLD following the criteria reported by Kleiner et al. [35]. Total lipid was extracted from liver samples using ethyl alcohol (g:v (mL) = 1:9), and the hepatic TG and TC contents were detected according to the protocol described in previous work [23].

### 4.5. Liver Lipidomic Analysis

A 1 mL volume of the extraction solvent (MTBE: MeOH =3:1, *v*/*v*) containing internal standard mixture was added to the liver tissue samples (50 mg). After homogenization, the mixture was vortexed for 1 min and then centrifuged at 12,000 rpm for 10 min. Then, 500 μL of the upper organic layer was collected and evaporated using a vacuum concentrator. The dry extract was reconstituted using 200 μL of mobile phase B (acetonitrile/isopropanol (10:90, *v*/*v*)) prior to analysis.

The lipid profile of the sample extracts was analyzed using a UPLC-ESI-MS/MS system (UPLC, ExionLC AD, MS, QTRAP^®^ System). Samples were analyzed on a Thermo Accucore™ C30 column (2.6 μm, 2.1 mm × 100 mm, City, CA, USA) with an injection volume of 2 μL and a flow rate of 0.35 mL/min, and the column temperature was set at 45 °C. The mobile phases consisted of a mixture of acetonitrile/water (60:40, *v*/*v*) (A) and a mixture of acetonitrile/isopropanol (10:90, *v*/*v*) (B); both liquid mixtures contained 0.1% acetic acid and 10 mmol/L ammonium formate. The solvent gradient program was set as follows: 0–2 min, 20–30% B; 2–4 min, 30–60% B; 4–9 min, 60–85% B; 9–14 min, 85–90% B; 14–15.5 min, 90–95% B, 15.5–17.3 min, 95% B; 17.3–20 min, 95–20% B. The qualitative and quantitative analysis of the lipid profile was carried out using multiple reaction monitoring analysis at Wuhan MetWare Biotechnology Co., Ltd. The analytical methods and detailed work parameters were performed following the description reported in the literature [36,37].

### 4.6. RNA Extraction and mRNA Expression Levels using Quantitative Real-Time PCR (RT-qPCR)

Total RNA extraction and cDNA synthesis were carried out as previously reported [22]. RT-qPCR was performed using a Bio-Rad CFX96 system (*Bio-Rad)*. The primer sequences are listed in Table 2. The expression of targeted mRNA level was normalized to β-actin.

### 4.7. Western Blots (WBs)

Hepatic protein extraction and WB were performed as previously described [23]. Briefly, hepatic total and nuclear proteins from the liver tissues were extracted using the total nuclear protein extraction kit and protein extraction kit obtained from Beyotime Biotechnology (Beijing, China). The protein samples were subjected to sodium dodecyl sulfate (SDS)-PAGE and then transferred to a polyvinylidene difluoride membrane (Millipore, USA). Thereafter, these membranes were blocked with 5%BSA at an ambient temperature for 2 h. Next, the samples were incubated with primary antibodies including NRF2 (1:800 dilution), HO-1 (1:1000 dilution), NQO1 (1:1000 dilution), GCLC (1:1000 dilution), histone H3 (1:2000 dilution), β-tubulin (1:2000 dilution), SCD-1 (1:1000 dilution), and SREBP-1 (1:1000 dilution), overnight at 4 °C. Then, the membranes were washed with TBST three times and incubated with a horseradish-peroxidase-conjugated secondary antibody (1:2000 dilution) for 2 h at room temperature. The immunoreactive bands were observed using a gel image analysis system using enhanced chemiluminescence (Advansta, California, USA).

### 4.8. Data Analysis

SPSS version 22.0 software was used to analyze statistical significance using one-way parametric analysis of variance (ANOVA) followed by Tukey’s post hoc test. The area under the curves (AUCs) of the GTT were analyzed by Origin 8.0 software. All data are shown as means ± standard deviation (SD) of the mean. *p <* 0.05 was regarded as a significant difference.

## 5. Conclusions

In conclusion, our work proved that NOB supplementation can improve dyslipidemia, serum inflammatory factors, hepatic steatosis, and oxidative stress in HFD-induced mice and can, therefore, attenuate the development and progression of NAFLD. The results indicate that these useful effects of NOB might be largely associated with a reduction in FFAs, DGs, TGs, and CEs in the liver. Taken together, our findings provide evidence that NOB ameliorates NAFLD by regulating liver lipid metabolites, regulating lipid-metabolism-related factors and the hepatic Nrf2 pathway. This study is beneficial for better understanding the ameliorative effects of NOB on NAFLD.

## Figures and Tables

**Table 1:** Composition of animal diets.

Class Description	Ingredients	Chow (g)	HFD (g)	LNOB (g)	HNOB (g)
Protein	Casein, Lactic, 30 Mesh	200.00	200.00	200.00	200.00
Protein	Cystine, L	3.00	3.00	3.00	3.00
Carbohydrate	Lodex 10	35.00	125.00	125.00	125.00
Carbohydrate	Sucrose, Fine Granulated	354.00	72.80	72.80	72.80
Fiber	Solka Floc, FCC200	50.00	50.00	50.00	50.00
Fat	Lard	20.00	245.00	245.00	245.00
Fat	Soybean Oil, USP	25.00	25.00	25.00	25.00
Mineral	S10026B	50.00	50.00	50.00	50.00
Vitamin	Choline Bitartrate	2.00	2.00	2.00	2.00
Vitamin	V10001C	1.00	1.00	1.00	1.00
Dye	Dye, Blue FD&C #1, Alum. Lake 35–42%	0.05	0.05	0.05	0.05
compound	NOB	0	0	0.77	1.54
Total		1055.05 g	773.85 g	774.62 g	776.16 g

**Table 2:** Primer sequences used for RT-qPCR.

Gene	Forward Primer (5’->3’)	Reverse Primer (5’->3’)
Nrf2	GCCTCCAAAGGATGTCAATCA	GCCTCACCTCTGCTGCAAGTA
NQO1	TGGCGTAGTTGAATGATGTCTT	TTCGGTATTACGATCCTCCCT
HO-1	CCACATTGGACAGAGTTCACAG	CCTCACAGATGGCGTCACTTC
GCLC	GCACATCTACCACGCAGTCAAG	CATCGCCTCCATTCAGTAACAAC
GSTA2	TGTCCTTCCCATAGAGGTCAT	TGCTTCACTACTTCAATGCCC
SREBP-1c	ATCCAAGGGCATCTGAGAACTC	ATCCAAGGGCAGTTCTTGTG
SCD-1	TCCTCCTTGGATTGTGTAGAAACTT	AATGTCAGAAGAAATCAGGTGGGTA
FAS	CTGAGATCCCAGCACTTCTTGA	GCCTCCGAAGCCAAATGAG
LXRα	TCAGAAGAACAGATCCGCTTG	CGCCTGTTACACTGTTGCT
PPARα	AGGCTGTAAGGGCTTCTTTCG	GGCATTTGTTCCGGTTCTTC
CYP7A1	AACAACCTGCCAGTACTAGATAGC	GTGTAGAGTGAAGTCCTCCTTAGC
CYP27A1	GCCTCACCTATGGGATCTTCA	TCAAAGCCTGACGCAGATG
β-actin	TGT CCA CCT TCC AGC AGA TGT	AGCTCAGTAACAGTCCGCCTAGA

## Data Availability

The authors declare that the data supporting the findings of this study are presented within the manuscript. Additional data sources are also available from the corresponding author upon reasonable request.
